# Gender Differences in Smoking and Self Reported Indicators of Health

**DOI:** 10.1186/1472-6874-4-S1-S7

**Published:** 2004-08-25

**Authors:** Susan Kirkland, Lorraine Greaves, Pratima Devichand

**Affiliations:** 1 Department of Community Health and Epidemiology, Faculty of Medicine, Dalhousie University, Halifax, Canada; 2 British Columbia Centre of Excellence for Women's Health, E311-4500 Oak Street, Vancouver, Canada; 3 Department of Community Health and Epidemiology, Faculty of Medicine, Department of Medicine, Dalhousie University, Halifax, Canada

## Abstract

**Health Issue:**

Smoking among Canadian women is a serious public health issue. Using the 1998–99 National Population Health Survey, this study examined underlying factors contributing to differences in prevalence of smoking among subgroups of women and men, and its effects on self-reported indicators of health.

**Key Findings:**

In Canada, 26.4% of women and 29.2% of men were classified as current smokers. Higher levels of education and income were associated with decreased odds of current smoking. Adjusting for all other factors, being an ethnic minority decreased the odds of current smoking for both men and women (OR:0.35, 99%CI:0.23–0.54; OR:0.13, 99%CI: 0.09–0.20 respectively). Single mothers had the highest odds of smoking (OR: 2.12, 99%CI: 1.28–3.51) when compared to married mothers with children under 25 years of age. Current women smokers and current and former men smokers were less likely to report very good or excellent health compared with never smokers (OR: 0.83, 99%CI: 0.70–0.98; OR: 0.49, 99%CI: 0.41–0.60; OR: 0.75, 99%CI: 0.63–0.90 respectively). Women who were current smokers had increased odds of needing health care and not receiving it (OR: 1.50, 99%CI: 1.10–2.05).

**Data Gaps and Recommendations:**

Key issues for Canadian women include an increased prevalence of smoking among young girls and the strong association between smoking and social and economic disadvantage. Tobacco control policies and programs must target high-risk groups more effectively. Of particular importance is the development of programs and policies that do not serve to reinforce existing inequities, but rather, contribute to their amelioration.

## Background

Smoking among Canadian women is a serious public health issue. Although historically men have smoked more than women, the decline in smoking prevalence among men has been much more pronounced over the last few decades: down from 61% to 25% among men from 1965 to 2001, as compared with a reduction in prevalence from 38% to 21% over this period among women. [1] The secular pattern of smoking places Canada in the fourth stage of the tobacco epidemic, along with other countries of the developed world such as the United States, the United Kingdom, Western Europe and Australia. [2] This stage is characterized by a decline in smoking prevalence among both men and women, accompanied by a peak and subsequent decline in deaths attributable to smoking among men. For women, however, the peak and subsequent decline in smoking-related deaths lags behind men by approximately 20 years. To date, there is no evidence to substantiate that this peak has been achieved among Canadian women.

Ongoing surveillance of smoking prevalence has been conducted in numerous Canadian national and provincial cross-sectional surveys. [3-7] Smoking prevalence is typically presented in sex-disaggregated, and to a lesser extent, sex- and age-disaggregated format. Similarly, smoking-related mortality outcomes and some morbidity outcomes, such as cancers, have been adequately captured by age and sex. However, we have only recently begun to acknowledge and understand smoking and its health effects from a gendered perspective. Whereas sex is biologically determined, gender is socially constructed and influences our roles in society, our formation of identity and the way in which others respond to us. [8,9] An analysis of smoking that incorporates social, cultural and economic factors can illuminate its differential impact on the lives of subgroups of women and men. In addition, there are many health consequences attributed to smoking, yet only the long-term consequences, such as lung cancer, heart disease and respiratory problems, are typically considered. These outcomes occur in older age groups and hold little relevance outside these age groups. More subtle health effects due to smoking may be particularly important to consider for young and middle-aged adult smokers because they are meaningful in the context of their daily lives. Health indicators that reflect intermediate health outcomes due to smoking, such as restriction of activities or use of health services, have rarely been emphasized.

The purpose of this paper, therefore, was to examine smoking prevalence and selected smoking-related health indicators for specific subgroups of women and men, incorporating socio-economic determinants of health to consider more fully the impact of gender. Based on identified gaps in the literature, the following research questions were posed: (1) What are the differences in prevalence of smoking among subgroups of women and men, based on determinants of health such as geographic location, age, income, education and ethnicity? (2) Are there differential effects of smoking on selected self-reported indicators of health for women and men? A summary of the literature is followed by an analysis of data from the NPHS.

### Smoking Demographics and Trends

Dramatic variations in rates and trends of smoking are evident for specific subpopulations of women in Canada. There has recently been a disturbing trend whereby smoking rates among teenaged girls exceeded smoking rates among teenaged boys for the first time. [10] Among girls aged 15 to 19, 25.1% reported being daily smokers in 1998–1999 and 26% in 2001, as compared with 18.5% and 20% respectively for boys in this age group. [5,6] Girls also started smoking at a younger age, 41% of girls aged 15 to 17 reporting having smoked their first cigarette before age 13 as compared with 29% of boys. [10] In the last decade, daily consumption increased for girls aged 15 to 19 from 11.5 cigarettes per day in 1990 to 12.7 cigarettes per day in 1999; this has decreased to 10.8 in 2001. [6,10]

The association found between smoking and social and economic disadvantage is in accordance with the tobacco epidemic model. There is a clear socio-economic gradient with cigarette smoking: higher prevalence among women who live in low-income households, have low-status jobs or are unemployed, are lone parents or divorced, and have low levels of education. [11,12] High prevalence rates of smoking among pregnant women are of particular concern. Pregnant smokers tend to be younger, have low levels of education, reside in poorer neighbourhoods, and are more likely to be single compared with their non-smoker counterparts. [13-20]

Aboriginal peoples have the highest rates of smoking in Canada. In 1997, 62% of First Nations people and 72% of Inuit were smokers compared with 29% of the general Canadian population. [21] The 1996 NWT Alcohol and Drug Survey showed that within the Northwest Territories, smoking prevalence was 44.2% and rates were similar among men and women, at 52.0% and 49.7% respectively. For those aged 15 to 24, the prevalence of smoking was 64.3% compared with 32.4% for this age group nationally. In Nunavut, overall smoking prevalence was 63.9%, with a prevalence of 77.9% for those aged 15 to 24; gender-specific rates for age groups were not reported. Equally alarming is that smoking rates among First Nations and Inuit are not decreasing: the prevalence of 62% reported from the First Nations and Inuit Regional Health Survey in 1997 was unchanged from the Statistics Canada estimate of 62% reported from the Aboriginal People's Survey carried out in 1991. [7]

Higher rates of smoking have also been observed in Francophone populations compared with Anglophone women. The most recent information available for the Canadian Francophone population comes from a report on the smoking behaviours of Canadians based on the 1996–1997 NPHS. [22] The prevalence of smoking was 35% among Francophones aged 15 and up, markedly higher than the Canadian average of 26%. Among Francophone women, the prevalence rate was 35% as compared with 36% among Francophone men and 24% among Anglophone women. Of all Francophone women in Canada, Quebec women had the highest smoking prevalence (38%). Between 1985 and 1995, smoking prevalence had dropped among Francophone women by 3%, as compared with a drop of 7% among men.

### The Health Effects of Smoking

The number of deaths due to smoking-related illnesses has increased in Canada over the last decade, with a much steeper increase seen in women than men. [23] This paradox, of increasing smoking-related morbidity and mortality in the face of declining prevalence, is a consequence of the steady increase in smoking prevalence among women in earlier decades. The impact on health is now evident in the rising incidence of cancers, heart disease and respiratory diseases among women. The number of deaths attributable to smoking has increased by 77% for women, from 9,009 in 1985 to 15,986 in 1996, whereas the number among men increased only slightly over a similar time period. [10] In 1996, the three leading causes of death in both men and women were cancer, heart disease and cerebrovascular disease, of which 21% was attributable to smoking. [23] Smoking is also known to increase the risk of lung cancer, which overtook breast cancer as the leading cause of cancer mortality among Canadian women in 1993. [10]

Adverse effects of smoking have been documented for the female reproductive system and health states unique to women such as pregnancy, breast-feeding and fetal health. Smoking during pregnancy has been found to decrease placental blood flow and has been associated with intrauterine growth retardation, increased rates of perinatal death, complications of pregnancy, and fetal anomalies such as cleft lip and palate. [24,25] On average, the infants of women who smoke have a lower birth weight, reduced length, smaller head circumference and reduced gestational age than the infants of non-smoking women. [15,18] A significant negative correlation between the number of cigarettes smoked and birth weight has been demonstrated. [15]

Smoking has also been implicated in the etiology of diseases unique to women, such as cervical and breast cancer, [26,27] and diseases of higher prevalence among women, such as osteoporosis. [28] Respiratory conditions such as asthma, bronchitis and emphysema are known to be adversely associated with smoking and are more prevalent among female than male smokers. [12,29] From 1979 to 1994 in the United States, age-adjusted mortality from chronic obstructive pulmonary disease (COPD) decreased by 17.1% among men but increased by 126.1% among women. Women also had a significantly higher hospitalization rate for COPD than men when amount of smoking was taken into consideration.

## Methods

Various surveys are available in Canada that examine smoking and tobacco use, such as CTUMS (Canadian Tobacco Use Monitoring Survey) and the General Health Survey. For the purposes of this chapter, NPHS data were used because more comprehensive information on social determinants of health were required for a gender analysis. A secondary analysis of existing cross-sectional data from the NPHS 1998–1999 was undertaken. [5] The methods of data collection for the NPHS have been reported in detail elsewhere [30] and are described in Appendix A. The third wave, completed in 1998–1999, was conducted by telephone.

### Statistical Analysis

For this report, analyses were limited to respondents aged 15 and over from the health component of the survey, weighted to represent approximately 24 million Canadians. For all analyses conducted, probability weights provided in the NPHS microdata files documentation were used to account for the sample design. For all reported proportions, the approximate coefficients of variation provided by NPHS were checked and found to be within the acceptable range (CV 0%-16.5%). Confidence intervals were calculated using the approximate CVs. For regression analyses, the probability weights were rescaled to an average value of one in order to improve the variance calculation. While this procedure does not take into account stratification or clustering of the sample design, it does take into account unequal probabilities of selection. [30] In addition, rather than using 95% confidence intervals, the more stringent 99% confidence intervals were reported. Maximum-likelihood multinomial polytomous regression and logistic regression were used for multivariate analysis with non-smokers as the referent group.

### Measures

All variables were based on pre-defined categories used in the NPHS. Additional detail regarding these variables is available in the NPHS documentation. [30]

Smoking status was characterized as never, current or former on the basis of questions about whether individuals smoked cigarettes daily, occasionally or not at all, at present or ever. Income adequacy is a measure incorporating total household income and the number of people within the household. This variable was collapsed from five categories to three categories for multivariate analysis: "low" and "low middle" categories were combined into "low," and "middle" and "upper middle" categories were grouped into "middle." Marital status was grouped into three categories based on the presence of a partner. Education was regrouped for multivariate analysis into three categories by combining secondary and some post-secondary education categories in the original variable.

Household type groupings reflect a relationship matrix derived in the NPHS. [30] For the multivariate analysis, six categories from the original NPHS variable were combined to form four categories: "couple with children < 25" included "couple with children < 25" and "couple with children < 25 plus others"; "couple with children > 25" included "couple with or without children > 25" and "couple alone"; the remaining two categories did not change. An overall functional social support scale, constructed for the Medical Outcomes Study (MOS), was used for the present analysis. It incorporated four subscales measuring tangible social support (range 0–16), affection (0–12), positive social interaction (0–16) and emotional/informational support (0–32).

Geographic location reflected the urban classification used in the NPHS, in which rural was defined by Enumeration Area classifications, and Census Metropolitan Area (CMA) designation was used to identify residents in Montreal, Toronto and Vancouver. Visible ethnic minority status was determined according to whether individuals identified themselves as "White" or "Other."

The outcomes considered were primarily health indicators reflecting the intermediate consequences of smoking rather than disease states. These outcomes were restriction of activities, self-rated health, sense of coherence (a self-rated measure of mental health), consultation with health professionals, and health care needed but not received. Number of chronic conditions was also considered. Restriction of activity was based on the question "Because of a long term physical or mental condition or a health problem, are you limited in the kind or amount of activity you do?" Self-rated health status was measured using the Health Description Index, in which respondents report their health as being poor, fair, good, very good or excellent. This measure was collapsed for the multivariate analysis into two categories "poor/fair/good" and "very good/excellent." The Sense of Coherence (SOC) scale was used in the NPHS as an indicator of mental well-being, incorporating aspects of comprehensibility, manageability and meaningfulness. Stephens et al. defined scores equal to and above the 75th percentile as indicating high SOC. [31] Following this method, a score of 70 in the NPHS 1998–1999 data indicated the 75th percentile, and a dichotomized variable was constructed for the multivariate analysis to reflect this (scores of 70 and above indicate high SOC; scores below 70 indicate low SOC).

## Results

### Socio-demographic Characteristics

**Figure 1:**
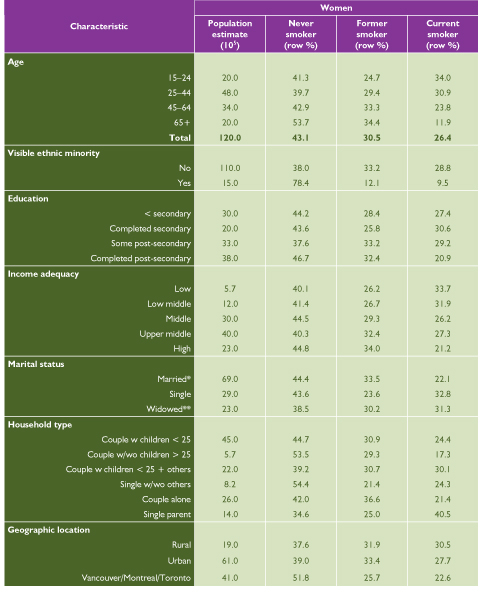
Socio-demographic Characteristics of Never Smokers, Former Smokers and Current Smokers: Women (NPHS 1998–1999)

**Figure 2:**
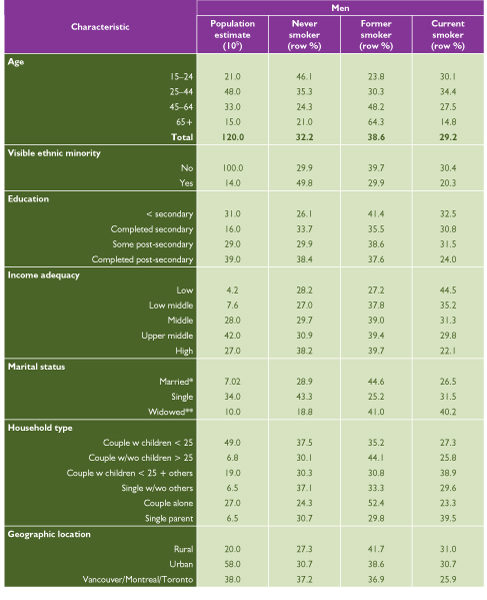
Socio-demographic Characteristics of Never Smokers, Former Smokers and Current Smokers: Men (NPHS 1998–1999)

The socio-demographic characteristics of never smokers, former smokers and current smokers are presented for women and men in Figures 1 and 2 respectively. Overall, 26.4% of Canadian women and 29.2% of Canadian men were classified as being current smokers. Among women, the highest proportion of current smokers (34.0%) was in the 15 to 24 age group, whereas for men the highest proportion (34.4%) was in the 25 to 44 age group. While the difference in proportions of current smokers differed only slightly for women and men aged 65 and older (11.9%: 95% confidence interval [CI] 9.7, 14.1, and 14.8%: 95% CI 12.0, 17.6 respectively), very different patterns of lifetime smoking history were noted: the majority of women of this age group had never smoked (53.7%: 95% CI 50.3, 57.1), whereas a much smaller proportion of men had never smoked (21%: 95% CI 17.8, 24.2). This is a function of historical gender differences in smoking uptake and cessation, as well as survival.

The prevalence of smoking among women and men of visible ethnic minorities was lower than among non-minority women and men; this difference was most pronounced among women (28.8%: 95% CI 27.4, 30.2 versus 9.5%: 95% CI 8.7, 10.3 for non-minority and minority women respectively). The prevalence of smoking according to level of education was similar among women and men, in that there were considerably lower rates of current smoking among those with the highest level of education (20.9%: 95% CI 19.0, 22.8, and 24.0%: 95% CI 21.9, 26.1, among women and men with post-secondary education respectively). A gradient was also noted for income adequacy, whereby smoking prevalence was lower for each increasing level of income adequacy. Interestingly, although the prevalence of smoking among women and men was similar in the high income adequacy category, the difference between women and men was more marked in the lowest income adequacy category, in which men were shown to have a higher prevalence of current smoking (44.5: 95% CI 37.3, 51.7 versus 33.7%: 95% CI 27.6, 40.0 among men and women respectively).

The prevalence of smoking varied by marital status and was lowest among those who were married (22.1%: 95% CI 20.5, 23.7 among women and 26.5%: 95% CI 24.9, 28.1 among men). When smoking was examined by household type, the highest prevalence of current smoking was seen among women and men heading lone-parent families (40.5%: 95% CI 36.8, 44.2 and 39.5%: 95% CI 33.8, 45.2, among women and men respectively).

**Figure 3:**
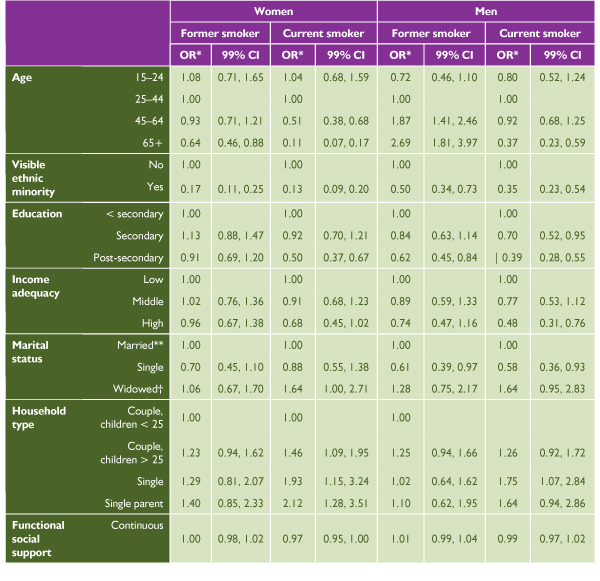
**Multivariate Association Between Socio-demographic Factors and Smoking Status (Former and Current Smoking Compared with Never Smoking), by Sex (NPHS 1998–1999)**. *OR = odds ratio; **Married/common law/partner †Widowed/separated/divorced

Figure 3 presents the results of multivariate analyses using polytomous regression to examine the association between socio-demographic factors and smoking status for women and men, adjusted for all other variables in the model. The modelling was done separately for women and men to determine the most parsimonious sex-specific models. The final model for women included two variables, household type and functional social support, that did not significantly contribute to the association for men. For the purposes of comparison, the same model was presented for both men and women. Associations for former smokers and current smokers compared with never smokers are presented for completeness, but the discussion addresses differences between current smokers and never smokers only.

Older age was associated with a decreased odds of current smoking for both men and women, with women aged 45 and up and men aged 65 and up less likely to be current smokers compared with those aged 24 to 44. Being of an ethnic minority also decreased the odds of current smoking for both men and women (odds ratio [OR]: 0.35, 99% CI 0.23, 0.54; OR: 0.13, 99% CI 0.09, 0.20 respectively). Women and men who had completed post-secondary education had a decreased odds of current smoking compared with those who had less than a secondary school education (OR: 0.50, 99% CI 0.37, 0.67; OR: 0.39, 99% CI 0.28, 0.55 respectively). Individuals in higher income adequacy categories were less likely to be current smokers, although for women this association achieved borderline statistical significance (women: OR: 0.68, 99% CI 0.45, 1.02; men: OR: 0.48, 99% CI 0.31, 0.76). Women in all household types other than those consisting of couples with children under 25 had increased odds of being current smokers, and single mothers had the highest odds (OR: 2.12, 99% CI 1.28, 3.51). For men, only single status increased the odds of being a current smoker (OR: 1.75, 99% CI 1.07, 2.84).

### Health Indicators

**Figure 4:**
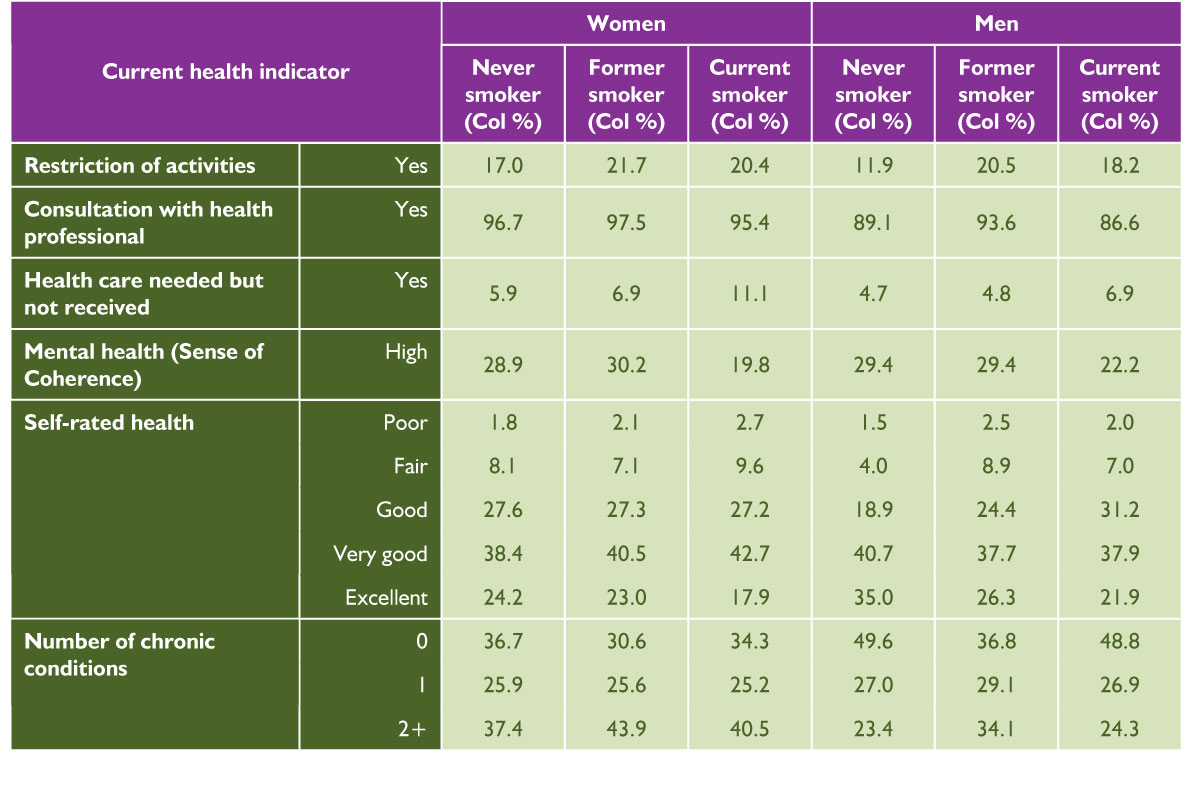
Current Health Indicators for Never Smokers, Former Smokers and Current Smokers, by sex (NPHS 1998–1999)

We then examined the proportions of never smokers, former smokers and current smokers who reported a variety of health indicators (Figure 4). Men who were current smokers, men who were former smokers and women who were current smokers were less likely to report very good or excellent health than never smokers, and less likely to obtain a high score on the SOC index than never smokers. Greater proportions of current and former smokers than never smokers indicated that a long-term limitation restricted their activities. The majority of individuals had consulted with a health care professional in the previous year, but men who were current smokers and those who were never smokers had done so the least (86.6%: 95% CI 87.7, 90.5 and 89.1%: 95% CI 85.0, 88.2 respectively). Almost double the proportion of women current smokers reported needing health care but not receiving it (11.1%: 95% CI 9.3, 12.9 and 6.9%: 95% CI 5.5, 8.3 among women and men respectively). Women who were current smokers and those who were former smokers reported the highest proportions of two or more chronic conditions (40.5% and 43.9% respectively, compared with 24.3% and 34.1% of men).

**Figure 5:**
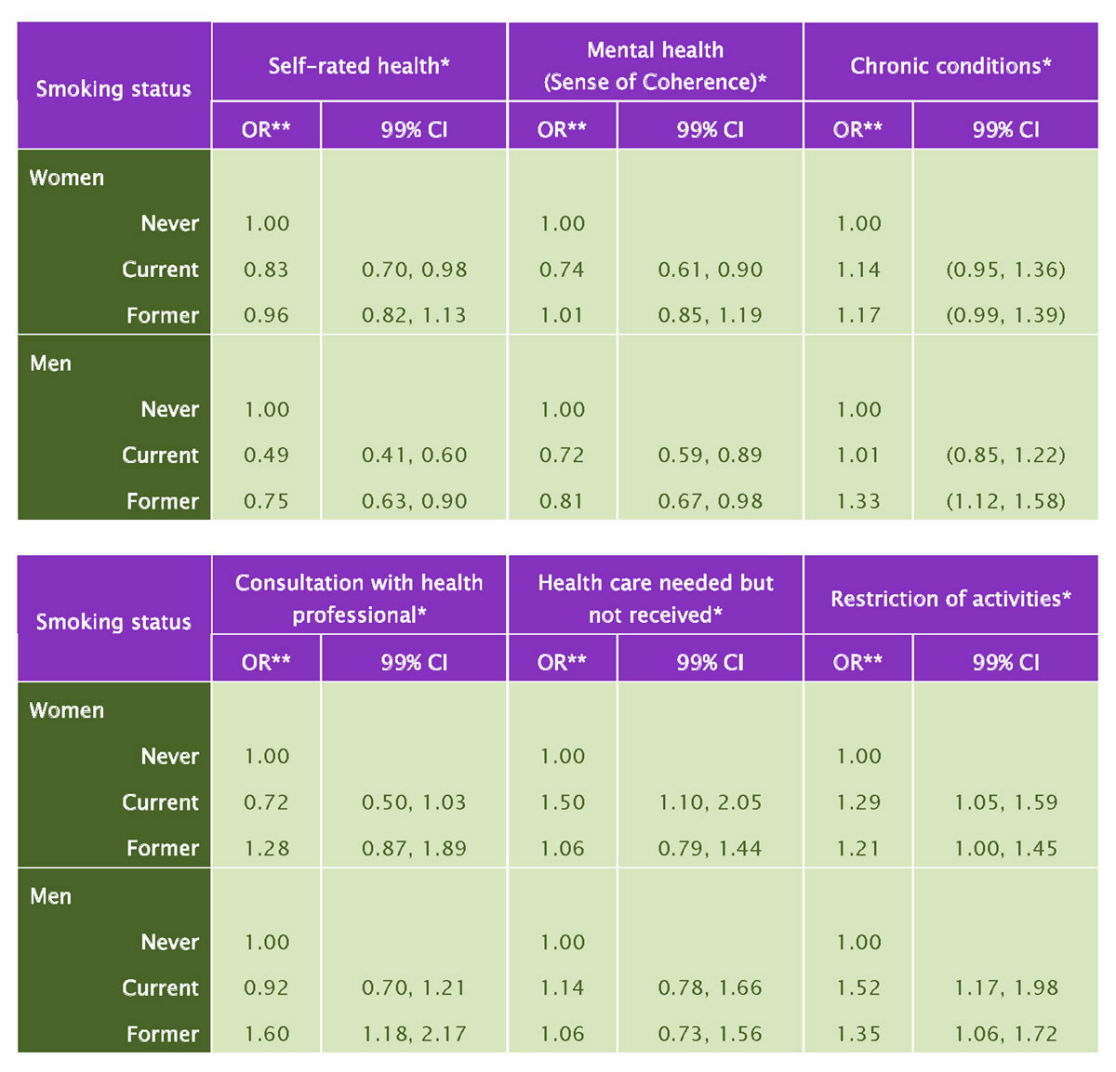
Multivariate Associations Between Smoking Status and Current Health Indicators, by Sex (NPHS 1998–1999) *Adjusted for age, marital status, visible ethnic minority, education, income adequacy, household type and functional social support **Odds ratio

Lastly, we examined the association between smoking status and each health indicator separately for men and women, adjusting for the previously considered socio-economic factors by means of logistic regression (Figure 5). Current women smokers and both current and former men smokers were less likely to report very good or excellent health compared with never smokers (OR: 0.83, 99% CI 0.70, 0.98; OR: 0.49, 99% CI 0.41, 0.60; OR: 0.75, 99% CI 0.63, 0.90, respectively). Likewise, women and men who were current smokers were less likely than their non-smoking counterparts to report high sense of coherence (OR: 0.74, 99% CI 0.61, 0.90; OR: 0.72, 99% CI 0.59, 0.89 respectively). Women who were current and those who were former smokers had similar odds of borderline statistical significance of having one or more chronic conditions (OR: 1.14, 99% CI 0.95, 1.36; OR: 1.17, 99% CI0.99, 1.39 respectively); only male former smokers had increased odds (OR: 1.33, 99% CI 1.12, 1.58). Former male smokers had increased odds of medical consultations (OR: 1.60, 99% CI 1.18, 2.17) compared with never smokers. Female current smokers had high odds of needing health care and not having received it (OR: 1.50, 99% CI 1.10, 2.05). Current smokers and former smokers had increased odds of restriction of activities compared with never smokers.

## Discussion

In this study, which used data from a population-based, national survey conducted in 1998–1999, we found smoking prevalence to be high for particular subgroups of both women and men that have previously been identified in the literature: younger age groups, lower income adequacy groups and lone-parent households. Despite the overall lower prevalence of smoking among women than men, the proportion of young women smokers exceeded that of men.

Age, marital status, ethnicity, education and income adequacy independently contributed to an association with current smoking for women and men. Age, ethnicity and marital status had strong associations for women, whereas education and income adequacy were strong factors for men. Interestingly, household type and functional social support contributed to the association with current smoking for women but not for men. The differences in these factors between women and men may reflect differences in life experiences in terms of social and family roles, work and care-giving. However, the fact that independent associations between socio-economic factors and smoking were seen for both women and men attests to their universal impact and may help to explain the high rates of smoking seen among subgroups that are disadvantaged in multiple aspects, such as Aboriginal populations.

In addition to considering the prevalence of smoking and the associated odds within subgroups of women and men, it is also important from a public health perspective to consider the population estimates within those subgroups. When the population estimates of lone parents are considered, there are many more women who are lone parents than men. This higher prevalence, combined with a strong magnitude of association between lone-parent status and smoking for women, implies that this group is at particularly high risk of smoking-related health problems.

In this study, associations between smoking and self-reported indicators of health were examined. Whereas the long-term effects of smoking on outcomes such as heart disease, cancer and respiratory conditions are well established, there are many more proximate effects of smoking that go unrecognized and that may differentially affect women and men. This study investigated six of these measures in order to explore these secondary, but important, differences. Women smokers reported greater restriction of activities, poorer mental health and more chronic health conditions than men who smoked. Although a greater proportion of women than men had consulted with a health professional in the previous year, twice as many women as men felt that they had health care needs that were not met. As compared with never smokers, independent associations were seen between current smoking and lower self-rated health, poorer mental health and greater restriction of activities for both women and men. Despite the higher prevalence of adverse health conditions noted among women as compared with men smokers, the higher background rates of health conditions among women often resulted in weak associations. This may reflect underlying gender differences in health; for example, women's perceptions of illness. However, it may also be the result of different patterns of smoking among men and women in terms of amount smoked and duration of use, which we were unable to account for.

It is relatively easy to compile information based on age and sex but more difficult to capture data from the complex range of factors that contribute to the impact that gender has on smoking and its health effects. While the present study made an attempt to capture some of these underlying factors, the difficulty of adequately measuring them remains. Examples of this include gender issues surrounding self-reported health, perceptions of the adequacy of access to health care and the gender-related nature of utilization of the health care system. Another issue is our lack of data, gender-related or otherwise, regarding ethno-cultural groups. We know from previous work among ethnic subpopulations in Canada and elsewhere that there are very different rates of smoking according to ethnic background and race, yet we have been unable to adequately document these aspects in population surveys in Canada. As a result, we are unable to provide sex- and gender-differentiated statistics to various minority groups that desperately seek information on their health status.

It is important to recognize that we used data from the 1998–1999 NPHS, a cross-sectional survey. This precluded us from assessing the long-term health sequelae of smoking, and it also meant that the associations documented cannot be interpreted as causal, since the appropriate temporal relation between smoking status and health indicators cannot be firmly established. Indeed, the associations observed may be due to self-selection effects rather than the effects of smoking per se. For example, it is possible that poor mental health in smokers may be the result of people with recurrent depression using smoking to achieve the stimulant effects of nicotine. Similarly, SOC may be a predictor rather than an outcome of smoking. It is also difficult to interpret the results for former smokers, as it is reasonable to expect that former smokers reflect two very different types: those who quit prior to a change in health status, and those who quit as a consequence of an adverse health outcome. Finally, by choosing to use the more stringent 99% confidence limits we may have missed some true associations.

In summary, smoking is one of the strongest modifiable risk factors for a host of health outcomes that contribute to female morbidity and mortality in Canada and worldwide. The contribution to the literature that this study provides is the control of potential, confounding socio-economic characteristics in a multivariable analysis examining smoking and smoking-related health outcomes for both women and men. Key issues for Canadian women include an increased prevalence of smoking among young girls and the strong association between smoking and social and economic disadvantage.

## Recommendations

The high prevalence of adverse intermediate health outcomes noted for women smokers is worthy of further investigation. Examining and acknowledging the importance of studying smoking and its health sequelae in a sex- and gender-differentiated manner is a valid starting point, but is clearly not enough. Further work remains to be done on the development of well-constructed socio-demographic and socio-economic health indicators that can be routinely collected and analyzed in population-based surveys to elucidate the impact of sex and gender on women's health in relation to smoking. For example, data that adequately capture the complexity of issues that women face in terms of occupation and employment status – balancing paid and unpaid work and caregiving roles – are likely to contribute to an understanding of smoking and smoking-associated health outcomes. The knowledge gained can then be used to inform the development of tobacco control policies and programs that may target high-risk groups more adequately and effectively. Of particular importance is the development of programs and policies that do not serve to reinforce existing inequities but, rather, contribute to their amelioration.

## Note

The views expressed in this report do not necessarily represent the views of the Canadian Population Health Initiative, the Canadian Institute for Health Information or Health Canada

## References

[B1] (2002). The National Strategy: Moving Forward. The 2002 progress report on tobacco control.

[B2] Lopez AD, Collinshaw NE, Piha T (1994). A descriptive model of the cigarette epidemic in developed countries. Tobacco Control.

[B3] Ferrence R, Stephens T (2000). Monitoring tobacco use in Canada: the need for a surveillance strategy. Chron Dis Can.

[B4] Kendall O, Lipskie T, MacEachern S (1997). Canadian health surveys, 1950–1997. Chron Dis Can.

[B5] Statistics Canada (2000). NPHS Cycle 3 (1998–1999), public use microdata files documentation.

[B6] Health Canada (1999). Canadian Tobacco Use Monitoring Survey (CTUMS) wave 2 Trends in smoking.

[B7] NWT Bureau of Statistics (1996). NWT Alcohol & Drug Survey: rates of use for alcohol, other drugs and tobacco.

[B8] Greaves L, Barr VJ (2000). Filtered policy: women and tobacco in Canada British Columbia Centre of Excellence for Women's Health.

[B9] Greaves L, Hankivsky O, Amaratunga C (1999). CIHR 2000: sex, gender and women's health The British Columbia Centre of Excellence for Women's Health: British Columbia Women's Hospital and Health Centre.

[B10] Health Canada (2001). Canadian Tobacco Use Monitoring Survey (CTUMS) wave 1 Summary of results.

[B11] INWAT (1999). Report of INWAT Europe Seminar on Women and Tobacco The International Network of Women Against Tobacco.

[B12] Chen Y, Dales R, Krewski D (1999). Increased effects of smoking and obesity on asthma among female Canadians: the National Population Health Survey, 1994–1995. Am J Epidemiol.

[B13] Najma JM, Lanyon A, Anderson M (1998). Socioeconomic status and maternal cigarette smoking before, during and after a pregnancy. Aust N Z J Public Health.

[B14] Muhajarine N, D'Arcy C, Edouard L (1997). Prevalence and predictors of health risk behaviours during early pregnancy: Saskatoon Pregnancy and Health Study. Can J Public Health.

[B15] Godel JC, Pabst HF, Hodges PE (1992). Smoking and caffeine and alcohol intake during pregnancy in the northern population: effect on fetal growth. Can Med Assoc J.

[B16] Mustard CA, Roos NP (1994). The relationship of prenatal care and pregnancy complication to birth weight in Winnipeg, Canada. Am J Public Health.

[B17] Dejin-Karlsson E, Hanson BS, Östergren P-O (1996). Psychosocial resources and persistent smoking in early pregnancy – a population study of women in their first pregnancy in Sweden. J Epidemiol Community Health.

[B18] Millar WJ, Chen J (1998). Maternal education and risk factors for small-for-gestational-age births. Health Rep.

[B19] Ruggiero L, Tsoh JY, Everett K (2000). The transtheoretical model of smoking: comparisons of pregnant and non-pregnant smokers. Addict Behav.

[B20] Davis RL, Tollestrup K, Milham S (1990). Trends in teenage smoking during pregnancy. Am J Dis Child.

[B21] Reading J (1999). The tobacco report: First Nations and Inuit Regional Health Surveys.

[B22] National Clearing house on Tobacco and Health (1999). Smoking behaviour of Canadians: National Population Health Survey highlights.

[B23] Makomaski Illing EM, Kaiserman MJ (1999). Mortality attributable to tobacco use in Canada and its regions, 1994 and 1996. Chron Dis Can.

[B24] Larsen LG, Clausen HV, Jonsson L (2002). Stereologic examination of placentas from mothers who smoke during pregnancy. Am J Obstet Gynecol.

[B25] Sastry BV (1991). Placental toxicology: tobacco smoke, abused drugs, multiple chemical interactions, and placental function. Reprod Fertil Dev.

[B26] Kuper H, Boffetta P, Adami H-O (2002). Tobacco use and cancer causation: association by tumor type. J Intern Med.

[B27] Moore TO, Moore AY, Carrasco D (2001). Human papillomavirus, smoking, and cancer. J Cutan Med Surg.

[B28] Holm K, Dan A, Wilbur J (2002). A longitudinal study of bone density in midlife women. Health Care Women Int.

[B29] Chen Y, Breithaupt K, Muhajarine N (2000). Occurrence of chronic obstructive pulmonary disease among Canadians and sex-related risk factors. J Clin Epidemiol.

[B30] Statistics Canada (1998). National Population Health Survey: health institutions public use microdata file – documentation.

[B31] Stephens T, Dulberg C, Joubert N (1999). Mental health of the Canadian population: a comprehensive analysis. Chron Dis Can.

